# Beyond social media: the era of generative AI and intelligent digital platforms in nephrology education

**DOI:** 10.1080/0886022X.2026.2658349

**Published:** 2026-04-22

**Authors:** Wisit Cheungpasitporn, Francesco Pesce, Charat Thongprayoon, Jing Miao, Muhammad Yasir Baloch, Karim Soliman, Jonathan Samuel Chávez Iñiguez

**Affiliations:** a Division of Nephrology and Hypertension, Mayo Clinic, Rochester, MN, USA; b AI Content Lead, Mayo Clinic Alix School of Medicine, Mayo Clinic, Rochester, MN, USA; c Division of Renal Medicine, Ospedale Isola Tiberina – Gemelli Isola, Rome, Italy; d Department of Translational Medicine and Surgery, Università Cattolica del Sacro Cuore, Rome, Italy; e Division of Nephrology, Washington University in St. Louis, St. Louis, MO, USA; f Division of Nephrology and Kidney Transplantation, University of Pittsburgh Medical Center, Pittsburgh, PA, USA; g Department of Nephrology, Antiguo Hospital Civil of Guadalajara ‘Fray Antonio Alcalde’, Guadalajara, Mexico

**Keywords:** Artificial intelligence in nephrology, generative AI, large language models, intelligent digital platforms, clinical reasoning, digital health

## Introduction

Over the past decade, nephrology education has been reshaped by social media, which enabled real-time global knowledge exchange, democratized teaching, and helped rebuild interest in a field long challenged by perceived complexity and declining trainee engagement [1–4]. Platforms such as Twitter/X, Facebook groups, YouTube-based lecture channels, and Instagram visual abstracts formed the backbone of the ‘Nephrology Social Media Renaissance’ [1–3,5]. They broadened access to expert insights, facilitated journal clubs, empowered early-career voices, and elevated nephrology’s presence in medical education [6–9].

However, the next transformation is far more profound. The rapid evolution of generative artificial intelligence (AI, large language models (LLMs), agentic learning systems, and intelligent digital platforms is creating an entirely new pedagogical era that is defined not by broadcast communication but by adaptive, interactive, and data-driven learning ecosystems [10–14]. These technologies are not confined to educational innovation alone but increasingly operate at the intersection of learning and clinical practice. They support a continuum in which knowledge acquisition, clinical reasoning, and patient care are dynamically integrated, with learning occurring alongside real-time clinical decision-making [15]. These tools do not merely supplement education; they fundamentally reshape how nephrologists learn, reason, and practice [16,17].

This editorial explores how nephrology education is transitioning beyond social media toward a paradigm where generative AI acts as a cognitive amplifier, personalized tutor, research copilot, and clinical reasoning partner. Importantly, this transition reflects a broader shift from information dissemination to workflow-integrated cognitive support that spans both education and patient care. It examines the opportunities, limitations, and strategic design considerations for building a robust, future-proof educational ecosystem in the era of intelligent digital platforms.

## How social media transformed nephrology and why it is no longer enough

### Achievements of the social media era

**Figure 1.**
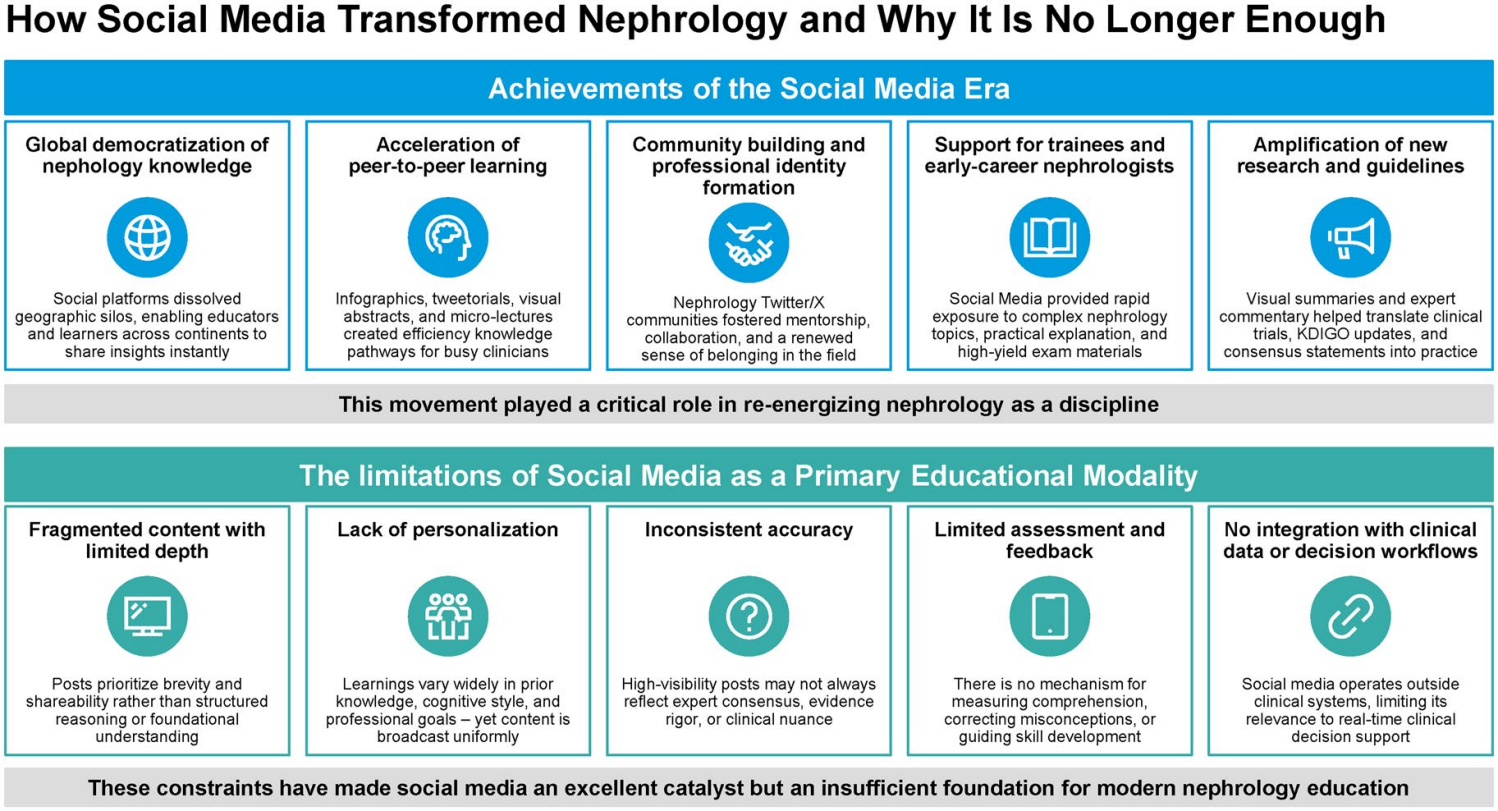
Social media in nephrology education: impact and limitations. Social media expanded access to nephrology education and community building but is limited by fragmented content, poor personalization, inconsistent accuracy, lack of assessment, and weak integration with clinical workflows, making it an effective catalyst but an insufficient standalone educational foundation.

Social media reshaped nephrology education in many meaningful ways (Figure 1) [5]. It opened global access to nephrology knowledge by dissolving geographic barriers and allowing clinicians, researchers, and trainees from every region to exchange ideas instantly [5,18]. This created an unprecedented level of knowledge democratization [2].

Peer to peer learning also accelerated. Infographics, tweetorials, visual abstracts, and short micro lectures became efficient tools that supported rapid comprehension for time constrained clinicians [6,7]. These formats allowed complex concepts to be communicated in a clear and visually structured manner, which helped increase engagement and retention [19,20].

The way information is disseminated on different social media platforms appeals to the inherent human capacity to quickly engage with what is visually attractive, with striking and intuitive images that can summarize an entire article. What we now know as the ‘Visual Abstract’ first appeared in 2016 in a surgery journal by Dr. Andrew Ibrahim, Creative Director of Annals of Surgery [21]. Today, journals and authors are convinced that creating an attractive Visual Abstract and posting it on a social network can disseminate it more quickly and efficiently.

Another major accomplishment was the formation of strong professional communities. Nephrology groups on Twitter and later on X fostered new mentorship relationships, collaborative initiatives, and a renewed sense of identity within the field [19,20]. Early career nephrologists and trainees in particular benefited from accessible explanations of difficult physiologic principles, practical clinical pearls, and high yield board preparation materials. In addition, social media became a powerful amplifier for new research [6,7]. Visual summaries, expert commentary, and real time conversations helped translate discoveries, guideline updates, and KDIGO recommendations into clinical practice more rapidly than traditional educational pathways [22,23].

These developments sparked a renewed enthusiasm for nephrology and expanded the discipline’s reach in ways that were not possible before the social media era.

### The limitations of social media as a primary educational modality

As influential as social media has been, its structure creates inherent limitations that constrain the depth and reliability of learning [5,24]. Content is often fragmented because posts favor brevity and shareability rather than stepwise reasoning or comprehensive explanation [5]. This can leave important clinical nuance unstated, particularly when topics require a full analytic framework [24].

Personalization is also limited. Learners differ in prior knowledge, cognitive style, and professional goals, yet content is broadcast uniformly to everyone [25]. Without adaptive delivery, the platform cannot account for individual learning needs or knowledge gaps [25].

Accuracy can be inconsistent [26]. Posts with high visibility may not always reflect expert consensus or strong evidence. Important details are often lost when complex topics are compressed for a general audience [27]. This increases the risk of oversimplification or misinterpretation [27].

Assessment is another major gap. Social media does not provide structured feedback, comprehension checks, or mechanisms to correct misconceptions [28–30]. Without these components, durable learning is difficult to achieve and skill progression is harder to evaluate [31]. Finally, social media remains disconnected from clinical data systems. It does not integrate with electronic health records, point of care applications, or real time decision support tools [31]. This limits its relevance to clinical workflow and reduces its ability to support precision medicine or context specific guidance.

It would be relevant for content creators to have academic and professional peer review. This would mitigate and modulate the limitations mentioned previously, resulting in more accurate and reliable information. We also believe it would be prudent to justify some academic comments made on social media with references and bibliographic sources whenever possible. This would provide readers with a reliable source of verification.

For these reasons, social media has served as a powerful catalyst for engagement and awareness but does not provide a reliable foundation for modern nephrology education. A new generation of intelligent digital platforms is now required to meet the evolving needs of clinicians, trainees, and health systems [10,12,13,17].

## Generative AI as the inflection point: from information access to cognitive amplification

### What makes generative AI transformational

Generative AI systems such as GPT based LLMs, Gemini AI, advanced multimodal architectures, agentic frameworks, and domain adapted nephrology models introduce capabilities that reshape how clinicians learn and reason [10]. Tools like NotebookLM add an additional layer by allowing users to build personalized knowledge bases from curated documents, creating highly tailored learning environments [10,11,32–34]. While these systems are widely discussed as educational tools, their role extends directly into clinical care by supporting real-time reasoning, data synthesis, and decision-making processes. In nephrology, this includes structured interpretation of laboratory trends, generation of differential diagnoses, and contextualization of complex physiologic data within patient-specific scenarios. In this sense, AI functions not only as an educational resource but as a cognitive support system embedded within clinical workflows.

**Figure 2.**
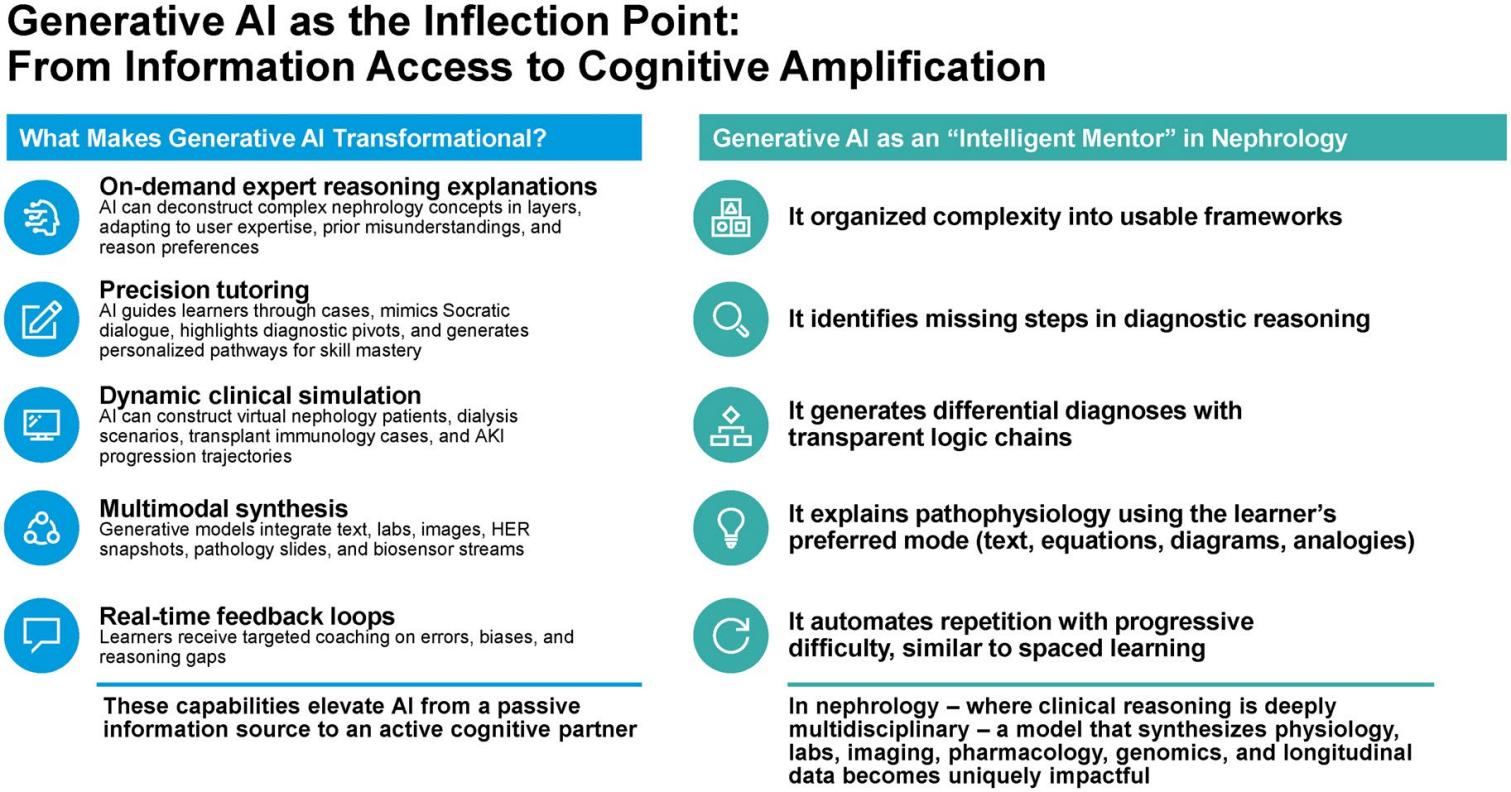
Generative AI as an inflection point in nephrology education: from information access to cognitive amplification. Generative AI enables expert-level reasoning, precision tutoring, simulation, multimodal synthesis, and real-time feedback, functioning as an intelligent mentor that transforms AI from an information source into an active partner in clinical reasoning.

These systems can provide on demand expert style explanations by breaking down complex nephrology concepts into progressive layers [13,14,16,35]. They can adjust to the learner’s background, the degree of prior misunderstanding, and the preferred reasoning style, which helps the learner progress with greater clarity and confidence (Figure 2) [16].

These platforms also function as precision tutors. A model can walk a clinician through a case, promote reflection through a Socratic style exchange, identify key diagnostic pivots, and create individualized pathways for skill development [13,14,16,35]. This supports mastery through guided reasoning rather than passive intake of information. The experience resembles the interaction with a dedicated mentor who adapts teaching strategy in real time.

Generative AI can also construct dynamic clinical simulations that include virtual nephrology patients, dialysis circuit scenarios, transplant immunology challenges, and trajectories of acute kidney injury [10,17,36,37]. These synthetic environments are safe, repeatable, and customizable. They make it possible for clinicians to practice decision making without risk and to explore uncommon or high risk situations that are rarely encountered in routine training [16].

Another major advantage is multimodal synthesis [16]. A single system can integrate text, laboratory data, imaging studies, electronic health record excerpts, biopsy pathology, point of care ultrasound clips, and even data streams from wearable biosensors [10,16]. This creates a unified analytic space that mirrors real world nephrology practice far more accurately than traditional educational materials. Learners can see how disparate data elements come together to shape a diagnostic or therapeutic strategy.

Generative AI also creates continuous feedback loops [10,13]. Learners receive targeted coaching when errors occur, when reasoning becomes inconsistent, or when biases influence interpretation [10]. This immediate corrective process deepens understanding and accelerates the development of expert level pattern recognition. With these capabilities, AI moves beyond the role of a reference source and becomes an active partner that supports cognitive growth and clinical reasoning at every step [17].

### Generative AI as an ‘intelligent mentor’ in nephrology

The greatest educational impact arises when generative AI behaves like a skilled mentor [13,14,16,35]. Instead of presenting isolated facts, it organizes complexity into coherent and usable frameworks [10,13]. It identifies missing steps in diagnostic reasoning and clarifies how one concept leads to the next. The model can build differential diagnoses with transparent reasoning chains that show exactly why each possibility is included or excluded [10,11,32–34].

Pathophysiology can be explained in the format that best suits the learner, whether through text, equations, diagrams, analogies, or integrated explanations [10,17]. Repetition can be automated with gradually increasing difficulty so that learners advance according to the principles of spaced and adaptive learning.

Nephrology places heavy demands on integrative reasoning because clinicians must combine physiology, laboratory interpretation, imaging, pharmacology, genomics, and longitudinal patient data [38,39]. A generative model that can synthesize these elements into a unified cognitive process becomes uniquely valuable. It supports learning in a way that mirrors real clinical decision making and provides a form of mentorship that is scalable, consistent, and endlessly adaptable [10,17].

## Intelligent digital platforms: the architecture of the post-social-media era

**Figure 3.**
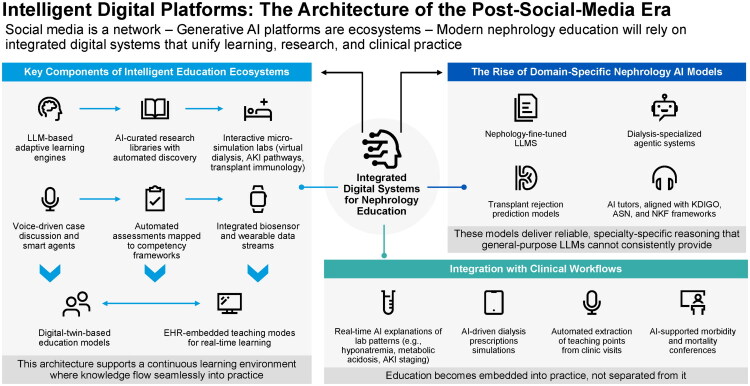
Intelligent digital platforms in post-social-media nephrology education. Post-social-media education relies on integrated digital ecosystems that unify learning, research, and clinical care through adaptive AI, simulations, automated assessment, digital twins, EHR integration, and domain-specific nephrology models.

Social media functions as a network [5,18]. In contrast, generative AI platforms operate as full ecosystems [10,14]. The next era of nephrology education will depend on intelligent digital systems that unify learning, research, and clinical practice within a single, continuously adaptive environment (Figure 3) [10,14,17]. These platforms extend beyond education alone, creating integrated environments in which clinical care, decision-making, and learning occur simultaneously.

### Key components of intelligent educational ecosystems

A high-performance nephrology platform rests on a set of interdependent capabilities that function as a unified learning ecosystem [10,40,41]. At its core are large-language-model adaptive learning engines that tailor instruction to individual learners, supported by AI-curated research libraries that surface emerging evidence [42,43]. Interactive micro-simulation environments for virtual dialysis, acute kidney injury pathways, and transplant immunology provide experiential learning that reflects clinical complexity [43]. Voice-based case discussion tools, powered by persistent conversational agents, extend faculty reach and reinforce clinical reasoning [43].

A critical component of these ecosystems is the integration of structured assessment and evaluation frameworks. Generative AI platforms can facilitate continuous self-assessment by providing real-time feedback on clinical reasoning, diagnostic accuracy, and decision-making processes. Through interactive case-based scenarios and adaptive questioning, learners can identify knowledge gaps and refine their understanding in a targeted and iterative manner.

Automated assessments are aligned with competency frameworks, and biosensor and wearable data streams introduce real-world physiologic context [44,45]. Beyond self-assessment, these systems enable systematic evaluation by tracking longitudinal performance across domains such as physiology, diagnostic reasoning, and therapeutic decision-making. AI-driven analytics can map learner progress to established competency frameworks, allowing educators to monitor development, identify areas requiring reinforcement, and tailor instruction accordingly.

Digital-twin educational models enable scenario testing and exploration of counterfactual outcomes [13,17]. When embedded within the electronic health record, dedicated teaching modes convert routine encounters into structured learning opportunities [10,46]. The integration of assessment within these platforms supports personalized learning pathways by continuously adjusting content difficulty and feedback based on performance, promoting mastery while maintaining alignment with clinical standards in nephrology education. Together, these components form an interconnected ecosystem that supports continuous learning within clinical workflows rather than functioning as isolated educational tools.

### The rise of domain-specific nephrology AI models

An important development in the field is the emergence of nephrology-specific AI systems designed to address the limitations of general-purpose models [46]. Nephrology-tuned LLMs are being trained to provide more consistent and contextually accurate reasoning across complex kidney-related decision pathways [46,47]. Dialysis-focused agentic systems are beginning to support prescription design, troubleshooting, and real-time optimization [48]. In transplantation, predictive models that integrate histopathology, molecular profiling, and clinical variables are advancing the assessment of rejection risk [49,50]. Educational tools are also evolving, with AI tutors increasingly aligned with KDIGO, ASN, and NKF frameworks to support standardized, competency-based training [10,17]. These developments reflect a broader shift toward specialty-specific intelligence, enabling performance and reliability that general-purpose models cannot consistently achieve [51]. As these models mature, their dual role in education and clinical decision support becomes increasingly evident, reinforcing the convergence of learning and patient care.

### Integration with clinical workflows

The distinguishing feature of intelligent educational platforms is their ability to merge seamlessly with clinical practice [42,52]. The integration of generative AI into clinical workflows represents a critical evolution beyond traditional educational use, extending into real-time clinical reasoning and decision support. These systems can synthesize complex laboratory trends, longitudinal patient data, and evolving clinical parameters into structured, interpretable insights, enabling clinicians to identify patterns, anticipate complications, and evaluate diagnostic or therapeutic options more efficiently.

In clinical practice, AI can generate real-time explanations of laboratory patterns, such as the differential evaluation of hyponatremia, the classification of metabolic acidosis, and the staging of acute kidney injury, while also simulating dialysis prescriptions with feedback on anticipated physiologic responses [42,52]. These systems can further extract key teaching points from clinical encounters, support case-based reflection, and strengthen morbidity and mortality discussions by reconstructing clinical trajectories and highlighting diagnostic uncertainty or cognitive bias [40,41,53].

This form of decision support is intended to complement, not replace, clinician expertise. When integrated appropriately, it enables more informed and timely decision-making while preserving physician judgment and patient-specific context. As a result, learning becomes embedded within routine care, occurring alongside clinical decision-making rather than as a separate activity [54,55].

## How generative AI supports different learner tiers

### Medical students

**Figure 4.**
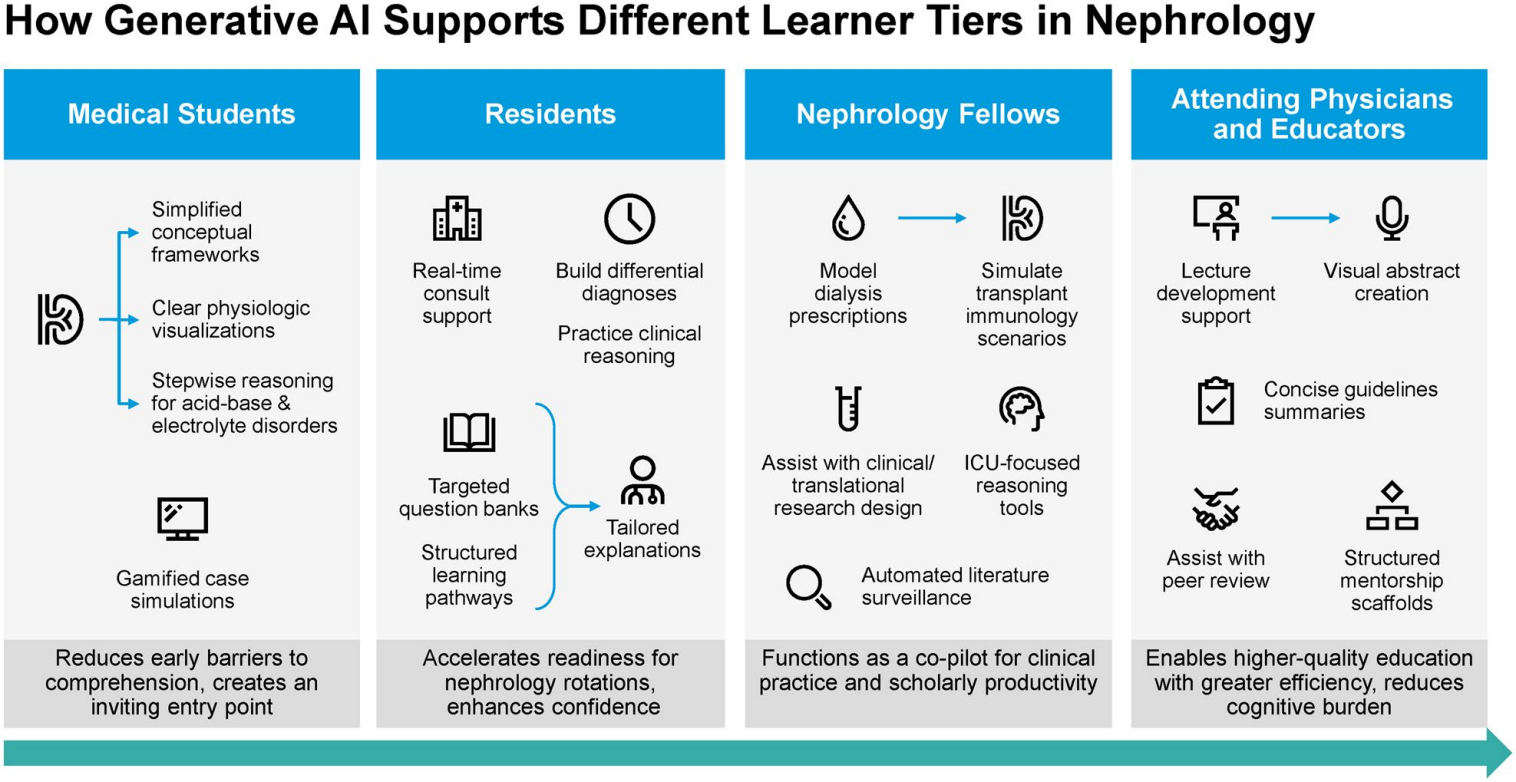
Generative AI support across learner tiers in nephrology. Generative AI supports all stages of nephrology training, from foundational learning for students to clinical, research, teaching, and mentorship support for residents, fellows, and attending physicians, enhancing efficiency across the educational continuum.

For medical students, generative AI lowers the threshold to engage with nephrology by providing simplified conceptual frameworks, clear physiologic visualizations, and stepwise reasoning for common acid–base and electrolyte disorders. It can also generate gamified case simulations that reinforce core principles in an accessible format [10,11,56]. These capabilities reduce early barriers to comprehension and create a more inviting entry point into the field (Figure 4) [11,56,57].

### Residents

Residents benefit from real-time support as they prepare for consults, build differential diagnoses, and practice clinical reasoning [16,56]. AI systems can generate targeted question banks, create structured learning pathways, and provide tailored explanations calibrated to the level of a senior resident [35,56,58]. This accelerates their readiness for nephrology rotations and enhances their confidence in managing kidney-related presentations.

### Fellows in nephrology

For subspecialty fellows, the impact is even more substantial. Generative AI can model dialysis prescriptions, simulate transplant immunology scenarios, and assist with the design of clinical or translational research studies [33,35,56,58]. It provides ICU-focused reasoning tools that map directly onto complex decision pathways and maintains automated surveillance of the literature to ensure continuous updates [35,56,58]. In this setting, AI functions as a true copilot for both clinical practice and scholarly productivity.

### Attending physicians and educators

Attending physicians and educators use generative AI to expand their teaching capacity while reducing cognitive burden [14,16,59]. The technology supports lecture development, visual abstract creation, and concise summaries of new guidelines. It also assists with peer review and provides structured mentorship scaffolds for trainees [35,56,58,59]. These capabilities enable faculty to deliver higher-quality education with greater efficiency.

## Opportunities created by generative AI in nephrology education

### Precision education

Generative AI enables a level of personalization that has not been possible in traditional instructional models [10,60]. Learning pathways can be adjusted to the individual’s baseline knowledge, specific clinical interests, preferred style of data interpretation, and prior clinical exposure [13,14,61,62]. Progress over time is monitored and used to refine subsequent content. This approach shifts nephrology education away from a uniform curriculum toward a genuinely precision-based learning model (Table 1) [14,62,63].

**Table 1.** Key opportunities and practical applications of generative AI in nephrology education.

Domain	Core contribution	Practical examples in nephrology
Precision education	AI personalizes instruction based on baseline knowledge, clinical interests, preferred reasoning style, and cumulative performance, shifting education toward precision learning.	Adaptive acid–base modules that adjust difficulty after each response. Personalized glomerulonephritis pathways tailored to a learner’s diagnostic strengths and weaknesses. Targeted micro-lessons on statistical interpretation for beginners or advanced fellows. Customized transplant immunology refreshers before clinic or call weeks.
Global workforce development	AI supports scalable, multilingual, and context-adapted training models, reducing reliance on large academic centers and addressing global nephrologist shortages.	Local-language teaching agents for AKI care in Southeast Asia or sub-Saharan Africa. Region-specific dialysis algorithms optimized for limited-resource settings. Virtual classrooms for rural or underserved areas with near-real-time mentorship. Clinical decision aids tuned to local epidemiology (e.g., higher rates of infectious GN).
Enhanced clinical reasoning development	AI improves diagnostic reasoning through structured prompts, improved pattern recognition, causal inference, and Bayesian updating, while also reducing cognitive bias.	Real-time, stepwise reasoning for hyponatremia or metabolic acidosis with explicit probability updates. Pattern matching for differentiating ATN vs. pre-renal AKI using evolving labs and vitals. Bias detection prompts during GN or transplant rejection evaluation. Interactive reasoning cases that update dynamically as new data ‘arrive.’
Research efficiency for trainees and faculty	AI accelerates idea generation, hypothesis refinement, data preprocessing, figure creation, code drafting, and manuscript development while operating within established and evolving ethical frameworks to ensure transparency, scientific integrity, appropriate attribution, and human oversight.	Rapid generation of multiple aims for AKI biomarker studies. Automated cleaning of dialysis datasets before statistical modeling. Drafting of tables, CONSORT-style diagrams, or PRISMA flowcharts. Starter templates for R or Python code for mixed-effects models. Auto-summarization of recent KDIGO or ASN guidelines for literature reviews, with human verification and ethical compliance.

AKI: Acute Kidney Injury; GN: Glomerulonephritis; ATN: Acute Tubular Necrosis; CONSORT: Consolidated Standards of Reporting Trials; PRISMA: Preferred Reporting Items for Systematic Reviews and Meta-Analyses; KDIGO: Kidney Disease: Improving Global Outcomes; ASN: American Society of Nephrology.

### Expansion of global nephrology workforce development

AI-driven platforms support scalable training in regions where nephrologist shortages remain a major barrier to kidney care [13,14,61,62]. They can provide tutoring in local languages, adapt algorithms to reflect regional patterns of disease and resource availability, and support decentralized or hybrid classrooms that operate independently of large academic centers [13,14,61,62]. These capabilities address disparities that earlier digital platforms, including social media, were unable to overcome.

### Enhanced clinical reasoning development

Generative AI strengthens clinical reasoning by guiding learners through structured diagnostic pathways [10,60]. It enhances pattern recognition, supports causal inference, and encourages explicit Bayesian updating as new data emerge. It can also highlight points where cognitive bias may influence interpretation [10,60]. These functions are particularly important for disorders that demand high-level synthesis, including acute kidney injury, glomerulonephritis, and transplant rejection.

### Research efficiency for trainees and faculty

Generative AI improves research productivity by accelerating idea development, hypothesis refinement, data cleaning, figure preparation, code generation, and manuscript drafting, thereby lowering barriers for early-career investigators [10,60]. It can rapidly generate multiple research aims for studies on acute kidney injury biomarkers, automate the cleaning of dialysis datasets before statistical modeling, and produce preliminary drafts of tables, CONSORT-style diagrams, or PRISMA flowcharts. It also offers starter templates for R or Python code to support mixed-effects modeling and can summarize new KDIGO or ASN publications to streamline literature reviews [13,14,61,62]. These efficiencies, however, must operate within established and evolving ethical frameworks that ensure transparency, protect scientific integrity, guard against undisclosed AI authorship, and preserve human responsibility for analytic decisions and interpretation [64].

## Risks, limitations, and the literacy required for safe adoption

The effective use of generative AI in nephrology requires a foundational level of AI literacy among educators and trainees [17]. Users must recognize common patterns of model reasoning failure, understand how to detect hallucinations, and remain aware of how bias can be propagated through training data or prompts [11,17,65]. They also need to distinguish between generative synthesis and true evidence-based inference, and they must understand the risks associated with inappropriate or undisclosed use of AI in scientific writing [65]. Without this literacy, even high-quality tools can be misapplied or misunderstood [14].

The safe use of generative AI also requires consideration of learner level and appropriate verification strategies. For medical students, AI-generated information should be treated as a preliminary resource rather than a definitive source. Learners should be encouraged to verify outputs using standard textbooks, high-quality peer-reviewed literature, and guidance from more experienced clinicians, including fellows, attending physicians, and educators. This approach supports the development of foundational knowledge while reinforcing critical appraisal skills. For residents, fellows, and practicing clinicians, generative AI can support more advanced clinical reasoning; however, reliance on AI-generated outputs without independent validation may introduce risks. Across all learner tiers, AI outputs should be cross-checked against established guidelines, primary literature, and clinical context to ensure accuracy and appropriateness.

Despite its transformative potential, generative AI has important limitations that must be acknowledged. One key limitation is the lack of true human empathy and relational understanding. While AI can generate contextually appropriate responses, it does not possess genuine emotional awareness, professional intuition, or the interpersonal skills required for effective patient-centered care. These elements remain central to clinical practice and cannot be replaced by automated systems. In addition, although generative AI can simulate a wide range of clinical scenarios, it cannot fully replicate the complexity of real-world nephrology practice. Clinical reasoning is shaped by dynamic patient conditions, uncertainty, multidisciplinary collaboration, and ethical or value-based considerations, all of which require human judgment and experience.

A fundamental limitation is that generative AI cannot replace human expertise, professional judgment, or accountability in clinical and educational decision-making. While AI systems can generate structured insights and recommendations, all outputs require interpretation, validation, and contextualization by clinicians. Without appropriate oversight, there is a risk of over-reliance on automated reasoning, which may reduce active human engagement in decision-making processes. In addition, the increasing presence of AI-generated or ‘semi-human’ content in digital environments introduces challenges related to authorship, credibility, and trust, particularly when distinctions between human-generated and AI-assisted contributions are unclear.

Rather than prescribing a fixed role for generative AI, a more appropriate approach is to establish guiding principles for its responsible integration. Reliability depends on the implementation of robust guardrails. These include the use of formal validation frameworks such as PROBAST-AI and TRIPOD-AI, transparent and reproducible prompt design, clear audit trails, and systematic human-in-the-loop evaluation to ensure that outputs remain clinically appropriate [66,67]. These mechanisms align AI-assisted education with established standards for methodological rigor and learner safety [67].

Ethical and professional considerations are equally important [17,68]. AI-generated content raises questions about proper attribution of ideas, maintenance of academic integrity, avoidance of over-reliance on automated reasoning, and the protection of patient privacy when clinical data are incorporated into educational tools [17,69]. Structural challenges must also be considered, including the digital divide, variability in infrastructure, and unequal access to advanced technologies, which may limit adoption, particularly in low-resource settings. Without deliberate efforts to address these disparities, AI-driven educational ecosystems risk reinforcing existing inequities in global nephrology training.

Generative AI should function as a supportive tool that augments clinical reasoning within transparent and validated frameworks and remains subject to continuous human oversight. In this role, it enhances clarity, efficiency, and learning while preserving clinician judgment, human expertise, and patient-centered care [16,35,69,70]. These principles provide a flexible foundation for responsible adoption as the technology continues to evolve.

## A strategic roadmap for nephrology education in the age of AI

### Build structured AI-enhanced curricula

Future nephrology education will require curricula that deliberately integrate AI capabilities into training [10,17]. Foundational modules in AI literacy should be paired with case simulations guided by LLMs and with digital twin learning environments that allow learners to explore physiologic and pathophysiologic trajectories [63,71,72]. Instruction should also incorporate AI-assisted interpretation of pathology and radiology studies as well as real-time clinical reasoning exercises that mirror decision-making at the point of care [17,73]. Together, these elements form a structured educational framework that reflects emerging practice realities (Figure 4).

### Develop specialty-specific AI tools

Progress will depend on the creation of nephrology-focused AI systems rather than continued reliance on general-purpose models [17,73,74]. Professional societies can play an important role by supporting the development of high-quality domain datasets, audit tools that monitor model behavior, reference-tuned models designed specifically for kidney disease, and benchmark tasks that allow objective evaluation of nephrology-oriented language models [17,73,74]. These efforts ensure that new tools align with clinical nuance and methodological rigor.

### Use AI to rebuild the nephrology pipeline

AI also offers an opportunity to strengthen the nephrology pipeline [10,17,61]. Earlier exposure to kidney physiology, delivered through adaptive and interactive modules, can make the field more accessible to learners who might otherwise find it intimidating. Just-in-time microlearning can reinforce key concepts at moments of need, and AI-enabled workflow support can reduce the cognitive load that often discourages trainees from pursuing nephrology [10,17,61]. These approaches position AI as an instrument for enhancing recruitment and long-term engagement.

### Create international AI education consortia

Sustained progress will require collaboration across institutions and regions [17]. International consortia focused on AI in nephrology education can establish harmonized standards, provide shared digital infrastructure, promote equity in access to advanced tools, and ensure that innovation remains both sustainable and globally inclusive [10,17,61]. Such collaborations create a foundation for consistent, high-quality training in the era of AI-enabled kidney care.

## Conclusion

Social media created a vibrant global nephrology community and helped democratize clinical insight, but the future of the field lies in intelligent learning ecosystems rather than broadcasting platforms. Generative AI, multimodal digital environments, and domain-specific intelligent assistants now offer an opportunity to reshape how nephrologists think, learn, teach, and innovate. The future of nephrology lies not in separating education from patient care, but in integrating both within intelligent, adaptive systems that support continuous learning and clinical decision-making. The coming era will be defined by precision learning, interactive simulation, continuous decision support, and AI-enabled cognitive augmentation. Generative AI offers the potential to function as both an educational catalyst and a clinical reasoning partner, enhancing the way nephrologists learn, think, and deliver care. When adopted responsibly, these developments can elevate the specialty by deepening conceptual understanding, expanding global access to high-quality training, and preparing clinicians for a rapidly changing landscape of kidney care. Realizing this potential will require careful implementation, ongoing evaluation, and a commitment to aligning technological innovation with patient-centered practice. Nephrology is poised for meaningful transformation, and the central task ahead is to guide this evolution with purpose, clarity, and a commitment to advancing patient-centered education and practice. Future implementation must remain aligned with evolving journal and institutional AI governance frameworks to ensure ethical, transparent, and responsible use.

## Data Availability

No new data were generated or analyzed in this editorial.

## References

[1] Stoneman S, Hiremath S. Twitter-based journal clubs: bringing critical appraisal to the social table. Semin Nephrol. 2020;40(3):264–272. doi: 10.1016/j.semnephrol.2020.04.004. 32560774

[2] Topf JM, Sparks MA, Phelan PJ, et al. The evolution of the journal club: from Osler to Twitter. Am J Kidney Dis. 2017;69(6):827–836. doi: 10.1053/j.ajkd.2016.12.012. 28233653

[3] O’Glasser AY, Jaffe RC, Brooks M. To tweet or not to tweet, that is the question. Semin Nephrol. 2020;40(3):249–263. doi: 10.1016/j.semnephrol.2020.04.003. 32560773

[4] Prasad C, Sanger S, Chanchlani R, et al. Engaging medical students and residents in nephrology education: an updated scoping review. J Nephrol. 2022;35(1):3–32. doi: 10.1007/s40620-021-01135-6. 34351594

[5] Shah SS, Zangla E, Qader MA, et al. Embracing the (r)evolution of social media and digital scholarship in pediatric nephrology education. Pediatr Nephrol. 2024;39(7):2061–2077. doi: 10.1007/s00467-023-06251-y. 38150027

[6] Larsen DM, Boscardin CK, Sparks MA. Engagement in free open access medical education by US nephrology fellows. Clin J Am Soc Nephrol. 2023;18(5):573–580. doi: 10.2215/CJN.0000000000000123. 36800537 PMC10278785

[7] Ramakrishnan M, Sparks MA, Farouk SS. Training the public physician: the nephrology social media collective internship. Semin Nephrol. 2020;40(3):320–327. doi: 10.1016/j.semnephrol.2020.04.012. 32560782

[8] Shaikh A, Patel N, Nair D, et al. Current paradigms and emerging opportunities in nephrology training. Adv Chronic Kidney Dis. 2020;27(4):291–296.e1. doi: 10.1053/j.ackd.2020.05.011. 33131641

[9] Raff AC. Great nephrologists begin with great teachers: update on the nephrology curriculum. Curr Opin Nephrol Hypertens. 2021;30(2):215–222. doi: 10.1097/MNH.0000000000000676. 33229909

[10] Miao J, Thongprayoon C, Craici IM, et al. How to incorporate generative artificial intelligence in nephrology fellowship education. J Nephrol. 2024;37(9):2491–2497. doi: 10.1007/s40620-024-02165-6. 39621255

[11] Boscardin CK, Gin B, Golde PB, et al. ChatGPT and generative artificial intelligence for medical education: ­potential impact and opportunity. Acad Med. 2024;99(1):22–27. doi: 10.1097/ACM.0000000000005439. 37651677

[12] Lawson McLean A. Constructing knowledge: the role of AI in medical learning. J Am Med Inform Assoc. 2024;31(8):1797–1798. doi: 10.1093/jamia/ocae124. 38812088 PMC11258398

[13] Cheungpasitporn W, Thongprayoon C, Kashani KB. Advances in critical care nephrology through artificial intelligence. Curr Opin Crit Care. 2024;30(6):533–541. doi: 10.1097/MCC.0000000000001202. 39248074

[14] Izquierdo-Condoy JS, Arias-Intriago M, Tello-De-la-Torre A, et al. Generative artificial intelligence in medical education: enhancing critical thinking or undermining cognitive autonomy? J Med Internet Res. 2025;27:e76340. doi: 10.2196/76340. 41183320 PMC12624298

[15] Cheungpasitporn W, Athavale A, Ghazi L, et al. Transforming nephrology through artificial intelligence: a state-of-the-art roadmap for clinical integration. Clin Kidney J. 2026;19(2):sfag004. doi: 10.1093/ckj/sfag004. 41704427 PMC12907560

[16] Triola MM, Rodman A. Integrating generative artificial intelligence into medical education: curriculum, policy, and governance strategies. Acad Med. 2025;100(4):413–418. doi: 10.1097/ACM.0000000000005963. 39705530

[17] Tangri N, Cheungpasitporn W, Crittenden SD, et al.; American Society of Nephrology (ASN) Artificial Intelligence (AI) Workgroup. Responsible use of artificial intelligence to improve kidney care: a statement from the American Society of Nephrology. J Am Soc Nephrol. 2026;37(4):881–890. doi: 10.1681/ASN.0000000929. PMC13065138 41201255

[18] Breu AC. From tweetstorm to tweetorials: threaded tweets as a tool for medical education and knowledge dissemination. Semin Nephrol. 2020;40(3):273–278. doi: 10.1016/j.semnephrol.2020.04.005. 32560775

[19] Cree-Green M, Carreau AM, Davis SM, et al. Peer mentoring for professional and personal growth in academic medicine. J Investig Med. 2020;68(6):1128–1134. doi: 10.1136/jim-2020-001391. PMC7418617 32641352

[20] Roberts MJ, Perera M, Lawrentschuk N, et al. Globalization of continuing professional development by journal clubs via microblogging: a systematic review. J Med Internet Res. 2015;17(4):e103. doi: 10.2196/jmir.4194. 25908092 PMC4424319

[21] Ramos E, Concepcion BP. Visual abstracts: redesigning the landscape of research dissemination. Semin Nephrol. 2020;40(3):291–297. doi: 10.1016/j.semnephrol.2020.04.008. 32560778

[22] KDIGO 2024 clinical practice guideline for the evaluation and management of chronic kidney disease. Kidney Int. 2024;105(4s):S117–s314. 38490803 10.1016/j.kint.2023.10.018

[23] KDIGO 2021 clinical practice guideline for the management of glomerular diseases. Kidney Int. 2021;100(4s):S1–s276. 34556256 10.1016/j.kint.2021.05.021

[24] Yao L, Li Y, Lian Q, et al. Health information sharing on social media: quality assessment of short videos about chronic kidney disease. BMC Nephrol. 2022;23(1):378. doi: 10.1186/s12882-022-03013-0. 36443741 PMC9703412

[25] Chen Y. Evaluation of the impact of AI-driven personalized learning platform on medical students’ learning performance. Front Med (Lausanne). 2025;12:1610012. doi: 10.3389/fmed.2025.1610012. 41020237 PMC12465117

[26] Borges do Nascimento IJ, Pizarro AB, Almeida JM, et al. Infodemics and health misinformation: a systematic review of reviews. Bull World Health Organ. 2022;100(9):544–561. doi: 10.2471/BLT.21.287654. 36062247 PMC9421549

[27] Desai AN, Ruidera D, Steinbrink JM, et al. Misinformation and disinformation: the potential disadvantages of social media in infectious disease and how to combat them. Clin Infect Dis. 2022;74(Suppl_3):e34–e39. doi: 10.1093/cid/ciac109. 35568471 PMC9384020

[28] Guckian J, Utukuri M, Asif A, et al. Social media in undergraduate medical education: a systematic review. Med Educ. 2021;55(11):1227–1241. doi: 10.1111/medu.14567. 33988867

[29] Scott N, Goode D. The use of social media (some) as a learning tool in healthcare education: an integrative review of the literature. Nurse Educ Today. 2020;87:104357. doi: 10.1016/j.nedt.2020.104357. 32032837

[30] Fouasson-Chailloux A, Daley P, Menu P, et al. Social media in health studies: a systematic review of comparative learning methods. Int J Environ Res Public Health. 2022;19(4):2205. doi: 10.3390/ijerph19042205. 35206401 PMC8871930

[31] Lowe-Calverley E, Barton M, Todorovic M. Can we provide quality #MedEd on social media? Trends Mol Med. 2022;28(12):1016–1018. doi: 10.1016/j.molmed.2022.08.002. 36008252

[32] Xu Y, Jiang Z, Ting DSW, et al. Medical education and physician training in the era of artificial intelligence. Singapore Med J. 2024;65(3):159–166. doi: 10.4103/singaporemedj.SMJ-2023-203. 38527300 PMC11060639

[33] Cheng Y, Zhu L. A review of ChatGPT in medical education: exploring advantages and limitations. Int J Surg. 2025;111(7):4586–4602. doi: 10.1097/JS9.0000000000002505. 40465793

[34] Mehta N, Benjamin J, Agrawal A, et al. Addressing educational overload with generative AI through dual coding and cognitive load theories. Med Teach. 2025 Aug 8:1–3. Epub ahead of print. doi: 10.1080/0142159X.2025.2543548. 40779508

[35] Boscardin CK, Abdulnour RE, Gin BC. Macy foundation innovation report part I: current landscape of artificial ­intelligence in medical education. Acad Med. 2025;100(9S Suppl 1):S15–s21. doi: 10.1097/ACM.0000000000006107. 40456178

[36] Garcia Valencia OA, Thongprayoon C, Miao J, et al. AI-generated explanations in kidney transplantation: accuracy vs. readability and implications for patient education. Front Artif Intell. 2026;9:1806516. doi: 10.3389/frai.2026.1806516. 41890628 PMC13012974

[37] Aiumtrakul N, Thongprayoon C, Kookanok C, et al. Quality assessment of large language model-generated prior authorization letters in nephrology. Front Digit Health. 2026;8:1767648. doi: 10.3389/fdgth.2026.1767648. 41852493 PMC12992280

[38] El-Achkar TM, Eadon MT, Kretzler M, et al. Precision medicine in nephrology: an integrative framework of multidimensional data in the kidney precision medicine project. Am J Kidney Dis. 2024;83(3):402–410. doi: 10.1053/j.ajkd.2023.08.015. 37839688 PMC10922684

[39] Eddy S, Mariani LH, Kretzler M. Integrated multi-omics approaches to improve classification of chronic kidney disease. Nat Rev Nephrol. 2020;16(11):657–668. doi: 10.1038/s41581-020-0286-5. 32424281

[40] Krishnan N. A hemodialysis curriculum for nephrology fellows using a blended learning approach: best of both worlds? J Nephrol. 2021;34(5):1697–1700. doi: 10.1007/s40620-020-00945-4. 33476037 PMC7818058

[41] Krishnan N, Brannan E, Hafler J, et al. Assessment of a hemodialysis e-curriculum in postgraduate nephrology education: a pilot mixed methods study. Clin Nephrol. 2022;98(5):247–255. doi: 10.5414/CN110778. 36149024

[42] William JH, Huang GC. How we make nephrology easier to learn: computer-based modules at the point-of-care. Med Teach. 2014;36(1):13–18. doi: 10.3109/0142159X.2013.847912. 24164578

[43] Lucas HC, Upperman JS, Robinson JR. A systematic review of large language models and their implications in medical education. Med Educ. 2024;58(11):1276–1285. doi: 10.1111/medu.15402. 38639098

[44] Stauss M, Htay H, Kooman JP, et al. Wearables in nephrology: fanciful gadgetry or Prêt-à-porter? Sensors (Basel). 2023;23(3):1361. doi: 10.3390/s23031361. 36772401 PMC9919296

[45] Madhvapathy SR, Cho S, Gessaroli E, et al. Implantable bioelectronics and wearable sensors for kidney health and disease. Nat Rev Nephrol. 2025;21(7):443–463. doi: 10.1038/s41581-025-00961-2. 40301646

[46] Parker MG. Nephrology training in the 21st century: toward outcomes-based education. Am J Kidney Dis. 2010;56(1):132–142. doi: 10.1053/j.ajkd.2009.11.029. 20418002

[47] Lemley KV. A brief review of some artificial intelligence methods in nephrology. Pediatr Nephrol. 2025 Oct 27. Epub ahead of print. doi: 10.1007/s00467-025-06995-9. PMC13139217 41145922

[48] Neri L, Zhang H, Usvyat LA. Artificial intelligence in kidney disease and dialysis: from data mining to clinical impact. Curr Opin Nephrol Hypertens. 2026;35(1):30–35. doi: 10.1097/MNH.0000000000001132. 41208283

[49] Loupy A, Sablik M, Khush K, et al. Advancing patient monitoring, diagnostics, and treatment strategies for transplant precision medicine. Lancet. 2025;406(10501):389–402. doi: 10.1016/S0140-6736(25)00195-3. 40614744

[50] Labriffe M, Woillard JB, Gwinner W, et al. Machine learning-supported interpretation of kidney graft elementary lesions in combination with clinical data. Am J Transplant. 2022;22(12):2821–2833. doi: 10.1111/ajt.17192. 36062389

[51] Hueso M, Vellido A. How artificial intelligence is transforming nephrology. BMC Nephrol. 2024;25(1):276. doi: 10.1186/s12882-024-03724-6. 39192232 PMC11348672

[52] Borbolla D, Gorman P, Del Fiol G, et al. Physicians perceptions of an educational support system integrated into an electronic health record. Stud Health Technol Inform. 2013;186:125–129. 23542982 PMC3745779

[53] Cook DA, Sorensen KJ, Nishimura RA, et al. A comprehensive information technology system to support physician learning at the point of care. Acad Med. 2015;90(1):33–39. doi: 10.1097/ACM.0000000000000551. 25374037

[54] McEvoy MD, Dear ML, Buie R, et al. Embedding learning in a learning health care system to improve clinical practice. Acad Med. 2021;96(9):1311–1314. doi: 10.1097/ACM.0000000000003969. 33570841 PMC8349926

[55] van Baalen S, Boon M, Verhoef P. From clinical decision support to clinical reasoning support systems. J Eval Clin Pract. 2021;27(3):520–528. doi: 10.1111/jep.13541. 33554432 PMC8248191

[56] Pham TD, Karunaratne N, Exintaris B, et al. The impact of generative AI on health professional education: a systematic review in the context of student learning. Med Educ. 2025;59(12):1280–1289. doi: 10.1111/medu.15746. 40533396 PMC12686775

[57] Koirala P, Thongprayoon C, Miao J, et al. Evaluating AI performance in nephrology triage and subspecialty referrals. Sci Rep. 2025;15(1):3455. doi: 10.1038/s41598-025-88074-5. 39870788 PMC11772766

[58] Lee P, Bubeck S, Petro J, et al. Limits, and risks of GPT-4 as an AI chatbot for medicine. reply. N Engl J Med. 2023;388(25):2400. 37342941 10.1056/NEJMc2305286

[59] Janumpally R, Nanua S, Ngo A, et al. Generative artificial intelligence in graduate medical education. Front Med (Lausanne). 2024;11:1525604. doi: 10.3389/fmed.2024.1525604. 39867924 PMC11758457

[60] Almansour M, Alfhaid FM. Generative artificial intelligence and the personalization of health professional education: a narrative review. Medicine (Baltimore). 2024;103(31):e38955. doi: 10.1097/MD.0000000000038955. 39093806 PMC11296413

[61] Zoccali C, Floyd L, Cseprekal O, et al. Artificial intelligence-driven nephrology: the role of large language models in kidney care. Am J Nephrol. 2025 Aug 28:1–15. Epub ahead of print. doi: 10.1159/000548208. 40875692

[62] Teo ZL, Thirunavukarasu AJ, Elangovan K, et al. Generative artificial intelligence in medicine. Nat Med. 2025;31(10):3270–3282. doi: 10.1038/s41591-025-03983-2. 41053447

[63] Loftus TJ, Shickel B, Ozrazgat-Baslanti T, et al. Artificial intelligence-enabled decision support in nephrology. Nat Rev Nephrol. 2022;18(7):452–465. doi: 10.1038/s41581-022-00562-3. 35459850 PMC9379375

[64] Miao J, Thongprayoon C, Suppadungsuk S, et al. Ethical dilemmas in using AI for academic writing and an example framework for peer review in nephrology academia: a narrative review. Clin Pract. 2023;14(1):89–105. doi: 10.3390/clinpract14010008. 38248432 PMC10801601

[65] Morgan DJ, Rodman A, Goodman KE. How physicians can prepare for generative AI. JAMA Intern Med. 2025;185(12):1407–1408. doi: 10.1001/jamainternmed.2025.4914. 41082216

[66] Collins GS, Dhiman P, Andaur Navarro CL, et al. Protocol for development of a reporting guideline (TRIPOD-AI) and risk of bias tool (PROBAST-AI) for diagnostic and prognostic prediction model studies based on artificial intelligence. BMJ Open. 2021;11(7):e048008. doi: 10.1136/bmjopen-2020-048008. PMC8273461 34244270

[67] Wang Y, Cheungpasitporn W, Ali H, et al. A practical guide for nephrologist peer reviewers: evaluating artificial intelligence and machine learning research in nephrology. Ren Fail. 2025;47(1):2513002. doi: 10.1080/0886022X.2025.2513002. 40620096 PMC12239107

[68] Li X, Yan X, Lai H. The ethical challenges in the integration of artificial intelligence and large language models in medical education: a scoping review. PLoS One. 2025;20(10):e0333411. doi: 10.1371/journal.pone.0333411. 41124146 PMC12543126

[69] Gin BC, O’Sullivan PS, Hauer KE, et al. Entrustment and EPAs for artificial intelligence (AI): a framework to safeguard the use of AI in health professions education. Acad Med. 2025;100(3):264–272. doi: 10.1097/ACM.0000000000005930. 39761533

[70] Garcia Valencia OA, Suppadungsuk S, Thongprayoon C, et al. Ethical implications of chatbot utilization in nephrology. J Pers Med. 2023;13(9):1363. doi: 10.3390/jpm13091363. 37763131 PMC10532744

[71] Zeng J, Qi W, Shen S, et al. Embracing the future of medical education with large language model-based virtual patients: scoping review. J Med Internet Res. 2025;27:e79091. doi: 10.2196/79091. 41232097 PMC12661241

[72] Sabanayagam C, Banu R, Lim C, et al. Artificial intelligence in chronic kidney disease management: a scoping review. Theranostics. 2025;15(10):4566–4578. doi: 10.7150/thno.108552. 40225559 PMC11984408

[73] Rosenberg ME, Anderson S, Farouk SS, et al. Reimagining nephrology fellowship education to meet the future needs of nephrology: a report of the American society of nephrology task force on the future of nephrology. Clin J Am Soc Nephrol. 2023;18(6):816–825. doi: 10.2215/CJN.0000000000000133. 36848491 PMC10278777

[74] Frehywot S, Vovides Y. An equitable and sustainable community of practice framework to address the use of artificial intelligence for global health workforce training. Hum Resour Health. 2023;21(1):45. doi: 10.1186/s12960-023-00833-5. 37312214 PMC10262492

